# Climate Predictions Accelerate Decline for Threatened *Macrozamia* Cycads from Queensland, Australia

**DOI:** 10.3390/biology1030880

**Published:** 2012-12-14

**Authors:** Melinda J. Laidlaw, Paul I. Forster

**Affiliations:** Queensland Herbarium, Department of Science, Information Technology, Innovation and the Arts, Brisbane Botanic Gardens, Mt Coot-tha Rd, Toowong 4066, Australia; E-Mail: Paul.Forster@science.dsitia.qld.gov.au

**Keywords:** climate change, conservation planning, cycads, distribution, *Macrozamia*, Maxent, Queensland, Australia, threatened species

## Abstract

Changes in the potential habitat of five allopatric species of threatened *Macrozamia* cycads under scenarios of increased ambient temperature were examined. A lack of seed dispersal, poor recruitment, low seedling survival, obligate pollinator mutualisms and continued habitat loss have led to extant populations being largely restricted to refugia. Models predict that the area of suitable habitat will further contract and move upslope, resulting in a reduced incidence within protected areas with increasing annual mean temperature. Areas of potential habitat for all five species are also predicted to become increasingly isolated from one another, further reducing the exchange between metapopulations and subpopulations, exacerbating existing threatening processes.

## 1. Introduction

The cycads (Cycadophyta: families Cycadaceae, Zamiaceae [1]) comprise dioecious, long-lived woody gymnosperms that have a perennial caudex or trunk and leaves that are shed and renewed over a period of several years. Cycads can be considered as ‘flagship species’ for conservation biology [2,3], as they have unusual life histories, are generally restricted in distribution, with over 50% of species threatened globally [4,5], and are of commercial interest to horticulture. Cycads as a group are considered to be in global decline due to climate change over time and their inability to outcompete most angiosperms [6]. 

Cycads are often referred to as the ‘dinosaurs of the plant world’ and certainly, the ancestral lineages of the current-day species were contemporaneous with dinosaurs, although it is likely that the extant genera (and component species) are relatively recently evolved [7,8]. Some authors consider that the surviving species of cycads are in an evolutionary dead-end and awaiting final extinction [6,9]; however, all species (and individual populations) have an origin, expansions and contractions of range, decline and an eventual extinction [10]. Many cycad species may be of recent origin, with species radiations since the Pleistocene (1.75 million–10,000 years ago) [11], although it has been postulated that the average length of time that a cycad species has existed for is about 54 million years [10]. 

Most species of cycads occur in populations that are often disjunct and with little evidence of genetic flow between them. Incipient inbreeding occurs within many populations [12,13,14,15,16]. It is perhaps unlikely that disjunct populations (even those that are geographically adjacent) of most cycad species behave as part of a dynamic ‘metapopulation’, where the local populations are regularly connected by dispersing individuals or have a flow of genetic material with the potential to recolonise or augment adjacent populations [17,18,19]. Rather, cycads fit a ‘regional ensemble’ model [20], with systems of essentially unconnected local populations persisting in an ill-defined mosaic of suitable and unsuitable habitat.

Most cycads appear to be characterised by dispersal-limited distributions [21], with sharp population boundaries, despite adjacent similar habitat [22,23]. Dispersal of seeds is local (less than 100 m from the parent), rather than long-distance (more than 100 m *sensu* [24]). Many of any resultant cycad seedlings are destroyed by events such as fire, competition or predation. Cycads are also generally absent from areas of disturbance or succession, where rapid establishment of individuals is an advantage [25]. This characteristic of reproductive failure drives a lack of contact between populations and probably has resulted in apparent speciation (reflected in morphological differences) via genetic drift rather than selection [26,27]. 

*Macrozamia* cycads are endemic to Australia, with forty-one species currently recognised and classified into two taxonomic sections [28,29,30]. Species of *Macrozamia* classified in *M.* section *Parazamia* are characterised by a subterranean caudex, leaves that are easily twisted off the stem and that have soft wool at the expanded base and leaflets that lack mucilage canals. Nearly all *Macrozamia* species from this section occur in few to numerous populations that vary in size from a handful of individuals to many thousands, and the majority of listed threatened (Endangered or Vulnerable) Macrozamias are from this group. 

**Figure 1:**
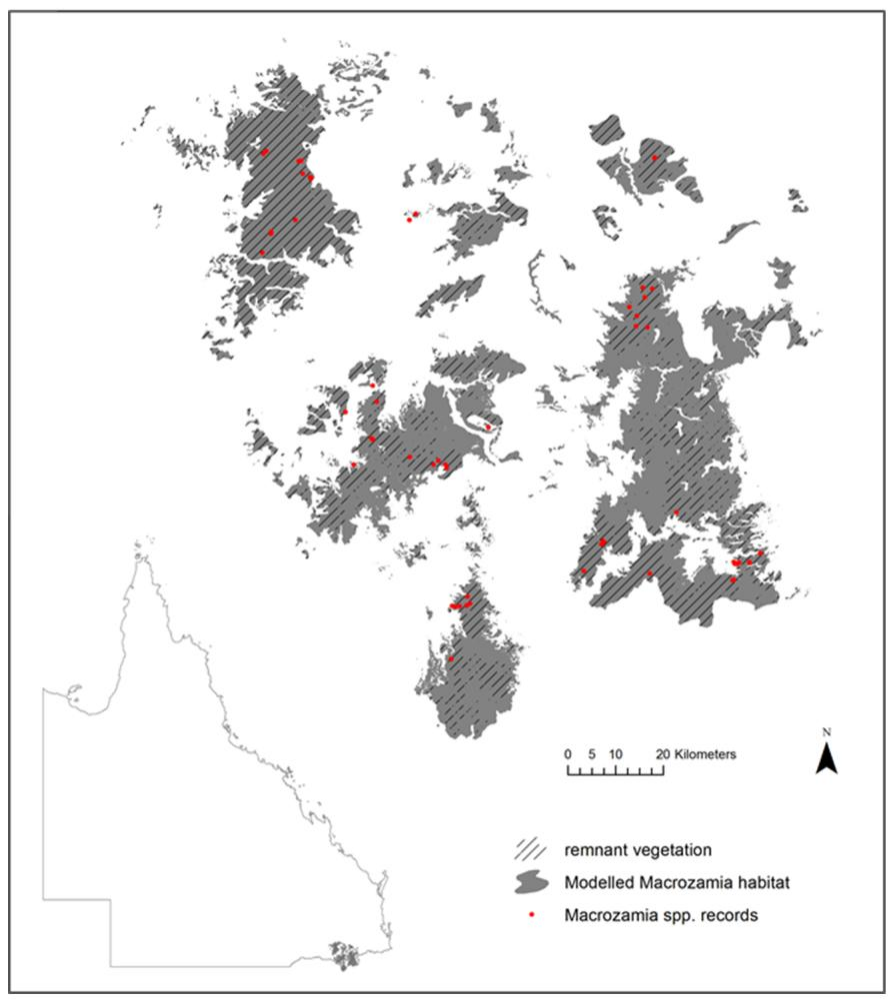
Current modelled range and species records of five *Macrozamia* species in the Darling Downs region of Queensland, Australia. Hatching indicates the extent of the current range covered by remnant vegetation.

In the Darling Downs district of south-east Queensland in eastern Australia (Figure 1), there are currently recognised five, morphologically similar species from *M.* section *Parazamia* (*M. cranei* D.L.Jones & P.I.Forst., *M. conferta* D.L.Jones & P.I.Forst., *M. machinii* P.I.Forst. & D.L.Jones, *M. occidua* D.L.Jones & P.I.Forst. and *M. viridis* P.I.Forst. & D.L.Jones). These five species occur allopatrically in generally consistent habitat types, but appear to share common reproductive traits of thermogenic male and female cones and a common pollinating beetle (the weevil *Tranes*) [31,32,33] and are probably biologically compatible if individuals from populations could interact genetically. They may be the result of a relatively recent speciation event [29], probably post-Pleistocene, as has been proposed for other cycads [11,27,34]. An alternative hypothesis is that they comprise five metapopulations of a single, highly variable ‘superspecies’ (referred to here as *Macrozamia* super spp.); either way, it is likely that they share a complex history of lineage interactions involving intermittent contact between populations, both within and between the currently recognised species. 

All five species are listed as threatened under Queensland’s Nature Conservation Act 1992 as either ‘Endangered’ (*M. cranei* and *M. viridis*) or ‘Vulnerable’ (*M. conferta*, *M. machinii* and *M. occidua*). Four are also listed federally under the Environment Protection and Biodiversity Conservation (EPBC) Act 1999 as ‘Endangered’ (*M. cranei*) or ‘Vulnerable’ (*M. conferta, M. machinii and M. occidua*). Two (*M. cranei* and *M. viridis*) are listed as ‘Endangered’, and the remaining three are listed as ‘Vulnerable’ on the IUCN red list. The current, fragmented distribution of the populations for these five species is an indication that they are largely in the ‘decline’ phase of species duration [10]. Many of the populations are very small (fewer than 100 adults) with little evidence of recruitment and may already be unviable in the long term. Individuals of these or similar cycads are long-lived (life span ranges of 60 to 1,500 years have been given for *Macrozamia* species [35,36]), resilient to fire and some forms of mechanical disturbance. In predicting future movements of species along with climate, species distribution models assume that species are in equilibrium with their environment [37,38]. This is difficult to assess if it is not known if a species is currently at its biophysical limits or if other factors are restricting its range [38]. In the case of the study species, which are long lived, exhibit low dispersal ability, but which are still reproducing *in situ*, we acknowledge that some extant adult individuals may have germinated under a different climate regime. These persistent ‘survivors’ seem capable of existing in small numbers; however, they are thought to be the last remnants of once healthy populations and may in reality be the ‘living dead’ in a biological sense, particularly if the obligate pollinators have been lost from individual populations.

## 2. Experimental Section

The species studied occur in the Darling Downs region of South-east Queensland (Figure 1), which experiences an annual average temperature of 11.9 °C–18.4 °C and an annual mean precipitation of 602–1,061 mm. Within a spatial context, these *Macrozamia* cycads exist on heavily weathered, nutrient poor soils derived from the remnants of a highly subdued landscape relief. The landscape is fragmented both from natural discontinuities in geology and associated ecosystems, compounded by land clearing for agriculture. Although much of the study region is covered by remnant vegetation (Figure 1), it is acknowledged that not all current or future land uses are compatible with the survival of *Macrozamia* cycads. Despite this, potential habitat has been modelled irrespective of land use with a view to its suitability for the restoration of *Macrozamia* habitat.

The populations of all five species have been extensively surveyed and the full extent of the species is believed to be known. Data used for potential habitat modelling represent specimen-backed records sampled from these populations and lodged with the Queensland Herbarium, vetted for taxonomic and spatial accuracy. Duplicate species presence records within an 83 m cell were excluded to reduce collection bias. The records for all five species were also pooled and modelled as a ‘superspecies’ (*Macrozamia* super spp.) comprising five metapopulations in acknowledgement of their putative close relationship [14,29,39]. 

The Maxent (3.3.3k) maximum entropy algorithm was used to model the potential habitat for all five *Macrozamia* species, as well as their ‘superspecies’. Records collected along roads were down-weighed to half the value of those collected away from roads to ensure the potential habitat models reflect each species’ known distribution, rather than the degree of sampling effort employed [40]. Seven uncorrelated predictor variables (pairwise Pearson correlations <0.83) considered to be functionally relevant to *Macrozamia* cycads were used to model all species. Four historic climatic variables, annual mean temperature, temperature seasonality (coefficient of variation), annual precipitation and mean moisture index of the lowest quarter moisture index, were derived using Anuclim 5.1 software [41] and an 83m digital elevation model (DEM). Topographic ruggedness was calculated from the DEM as the range in elevation covered by the eight cells surrounding each focal cell. Pre-clearing broad vegetation group (1:1M scale) and land zone, a classification of substrate and geomorphology, were also used as categorical predictor variables. 

Maxent models were trained on the predictor variables describing the observed historical base climate, then projected to predictor variables, where annual mean temperature was replaced with modelled future annual mean temperature values for the years 2030, 2050, 2070 and 2100 under two different emission scenarios. The A1B emission storyline describes a mid-line, ‘business as usual’ scenario of global economic growth, low population growth and a move towards a balancing of fossil fuel use with other sources of energy [42]. It assumes a moderate rate of global warming of approximately 2.6 °C with a doubling of atmospheric carbon from 280–560 ppm [42]. The second emission scenario used, A1FI, assumes a higher rate of global warming of 4.2 °C for a doubling of CO2 from 280–560 ppm and describes a world of increasing globalisation, economic growth and fossil fuel use [42]. For both scenarios, the Max Planck: ECHAM5/MPI-OM global climate model was selected for the prediction of future temperature conditions, as it produces a moderate annual warming response in comparison to other climate models [42]. In general, this model describes an increase in annual mean temperature across all of Australia, but with smaller increases along the southern coast [42].

**Figure 2:**
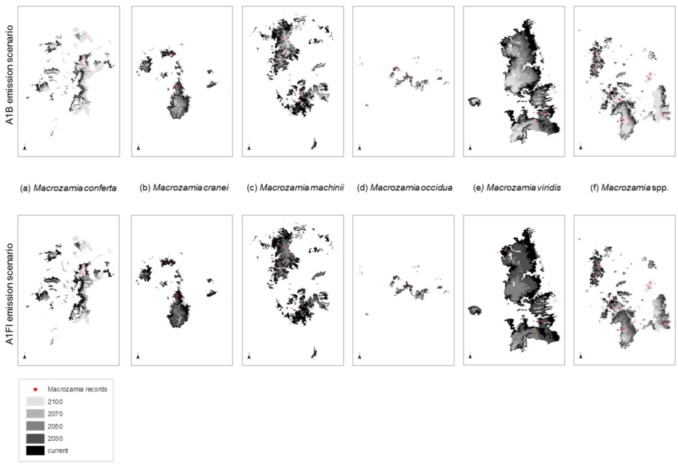
Predicted changes in the range of Darling Downs Macrozamia cycads with increasing annual mean temperature under A1B and A1FI emission scenarios **(a)** *M. conferta*. **(b)** *M. cranei*. **(c)** *M. machinii*. **(d)** *M. occidua*. **(e)** *M. viridis* and **(f)** *Macrozamia* super spp.

A mask was created for each species by buffering a minimum convex hull incorporating all records by 200 km. A 200 km buffer has been shown by VanDerWal *et al.* [43] to produce a model that both generalises well across a range of environmental conditions and does not over-inflate predicted distributions at the expense of finer environmental gradients. Maxent uses the masks to restrict the selection of background points (or pseudoabsences) to the region of species [43], ensuring that the model reflects the distribution of a species rather than the distribution of an environmental predictor [40]. Ten thousand random background samples were selected within the mask. Clamping was applied to restrict predictor values outside of the masked area to the range of values within the masked area [44]. Models were fitted using linear, quadratic and hinge features. A ‘tenth percentile training presence’ threshold was applied to all species to modify continuous logistic (0-1) Maxent outputs to a binary map of potential species distribution. This is a conservative threshold, which may lead to the occasional omission of presence records (e.g., Figure 2e). A majority filter algorithm was applied to both remove ‘orphan’ cells and smooth the margins of the distribution. 

Due to their restricted geographical range, only small numbers of presence records were available (Table 1), and as such, no data were quarantined for model testing. Instead, the accuracy of the distribution models was assessed via the area under the receiver operator curve (AUC). AUC indicates the ability of a model to distinguish between presence and absence [37]. The modelled AUC was compared to the 95% confidence interval upper limit AUC produced from 1000 null models of randomly selected sites chosen without replacement from within the bounds of a 200 km mask for each species [45]. 

**Table 1:** Contribution of annual mean temperature and temperature seasonality to each *Macrozamia* model, model AUC value and 95% confidence interval (CI) AUC values of the randomly drawn null-models (n = 1,000).

Species	n	Mean annual temperature permutation importance (%)	Temperature seasonality permutation importance (%)	Modelled AUC	95% CI AUC
*Macrozamia conferta*	12	0	0.9	0.992	0.935
*Macrozamia cranei*	13	54.6	43.2	0.993	0.938
*Macrozamia machinii*	18	58.4	10.6	0.993	0.899
*Macrozamia occidua*	5	88	8.2	0.998	0.995
*Macrozamia viridis*	11	89.3	3.7	0.994	0.983
*Macrozamia* super spp.	59	60.5	31.6	0.982	0.829

## 3. Results and Discussion

All species models performed significantly better (95% CI) than random when compared to 1000 null models of randomly selected locations from masks used for background point selection (Table 1). For four of the five species examined, and at the ‘superspecies’ level (*Macrozamia* super spp.), annual mean temperature (54.6–89.3% permutation importance) and temperature seasonality (3.7–43.2% permutation importance) were found to be limiting factors. Together, these two variables contributed 69–97.8% (mean 89.6% ± SE 5.26%) of the permutation importance, demonstrating the sensitivity of a majority of this group to temperature perturbation. The exception, *M. conferta,* is not driven by temperature (Table 1). Instead, it is limited by land zone (65.8%) and broad vegetation group (24.6%). Its current distribution is restricted to eucalyptus forest/woodland on poor soils of either silty loam or shallow rocky skeletal soils [14,39]. 

**Figure 3:**
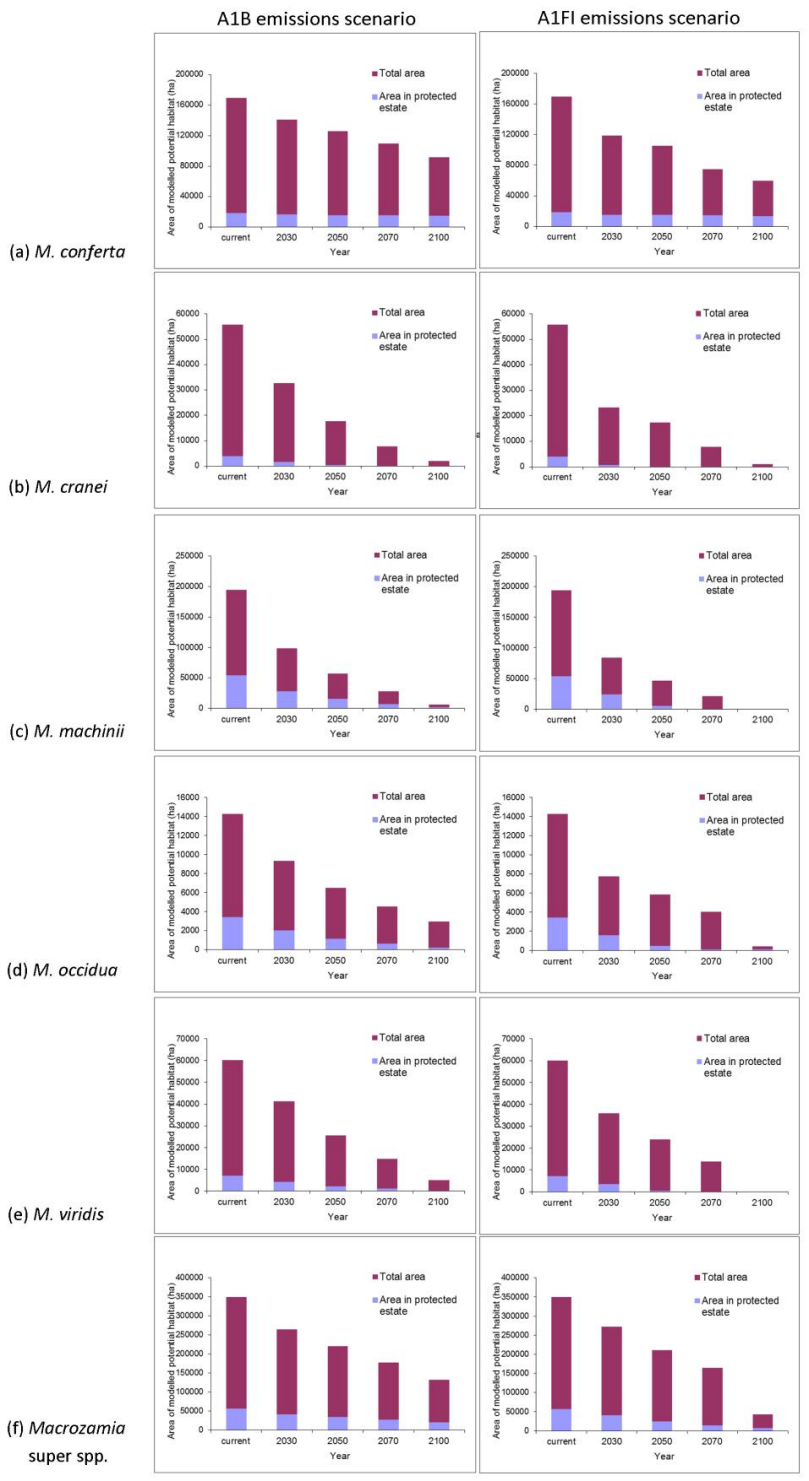
Predicted changes in the total area of potential habitat and area within protected areas of Darling Downs *Macrozamia* cycads and with increasing annual mean temperature under A1B and A1FI emission scenarios **(a)** *M. conferta*, **(b)** *M. cranei*, **(c)** *M. machinii*, **(d)** *M. occidua*, **(e)** *M. viridis* and **(f)** the ‘superspecies’ *Macrozamia* super spp.

The ranges of all species, as well as the ‘superspecies’, are predicted to become increasingly restricted within Queensland with increasing annual mean temperature (Figure 2). Range reduction is more pronounced under the A1FI emissions scenario in all cases. The area of potential habitat continually reduces with increasing annual mean temperature with two species, *M. machinii* and *M. viridis* having no potential habitat modelled at 2100 under the A1FI emissions scenario (Figure 3). Under both the A1B and A1FI emission scenarios, the range of *M. conferta* is predicted to become increasingly restricted around current presence records (Figure 2a) and for this area to remain as potential habitat up until at least the end of the century. The current distribution of this species may represent refugial populations restricted to this area under past climatic perturbations. The apparent dispersal limited distribution and the biological features that result in these traits appear to have historically ‘imprisoned’ each population within their refugial area. This is further compounded by the lack of suitable habitat in adjacent areas. Hence, the projected reduction in suitable habitat area paints a dire future scenario for this particular species. 

The area of potential habitat found within the current protected area estate is predicted to decline over time for most species (Figure 3). This situation is particularly serious for *M. cranei*, as none of its known populations are currently protected within reserves. Approximately 8% of this species’ modelled potential habitat is currently afforded some protection in National Park and various State Forests; however, this is predicted to decline to less than 5% of its potential distribution by mid-century (Figure 3b). Remnant vegetation on private lands where the species is currently found could be considered for greater protection. Uniquely, the modelled range of *M. conferta* within protected areas remains largely consistent, despite the reduction in overall area with increasing annual mean temperature (Figure 3a). This indicates that the current location of protected areas will remain important for this species into the future. 

**Figure 4:**
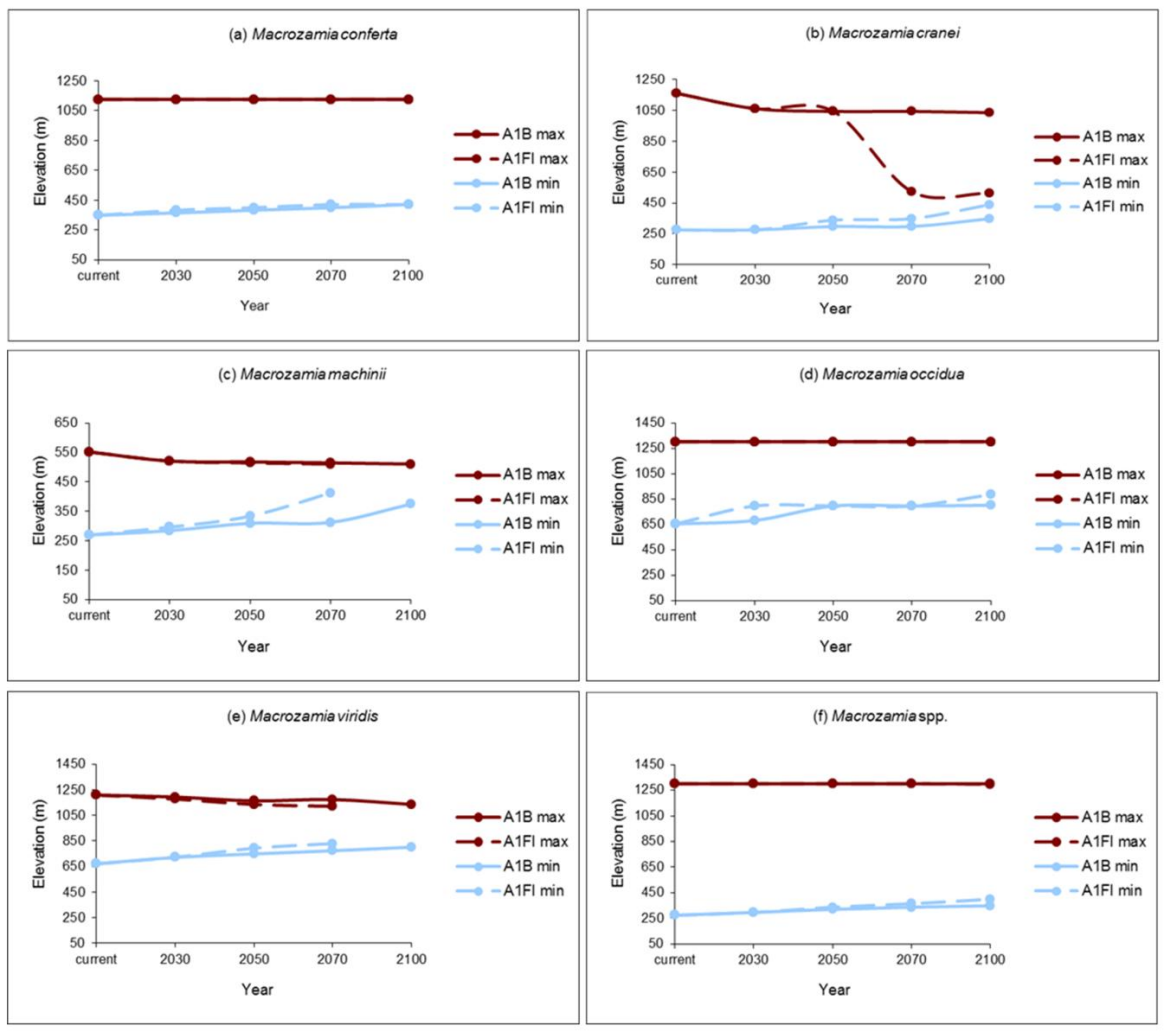
Maximum (max) and minimum (min) elevations of potential habitat ranges modelled for Darling Downs *Macrozamia* cycads with increasing annual mean temperature under A1B and A1FI emission scenarios **(a)** *M. conferta*, **(b)** *M. cranei*, **(c)** *M. machinii*, **(d)** *M. occidua*, **(e)** *M. viridis* and **(f)** *Macrozamia* super spp.

The elevation range of suitable potential habitat restricts with increasing annual mean temperature for all species (Figure 4). The upper elevational range limits for *M. conferta*, *M. occidua* and the ‘superspecies’ (*Macrozamia* super spp.) remain constant over time, while the lower limit of their distribution is predicted to rise under both the A1B and A1FI emission scenarios. *Macrozamia cranei*, *M. machinii* and *M. viridis* are predicted to experience a restriction towards the middle of their current elevation range that is more pronounced under the A1FI emissions scenario. This may be extremely pronounced in the case of *M. cranei* (Figure 4b).

Although we have only examined possible *in situ* upslope movement for these species, possible range movement south across the state border into New South Wales should be considered, where the related *M. plurinervia* occurs [14]. The lack of dispersal ability, coupled with extremely slow generational turnover, are likely biological traits preventing these species from shifting or expanding spatially at any substantial rate. 

**Figure 5:**
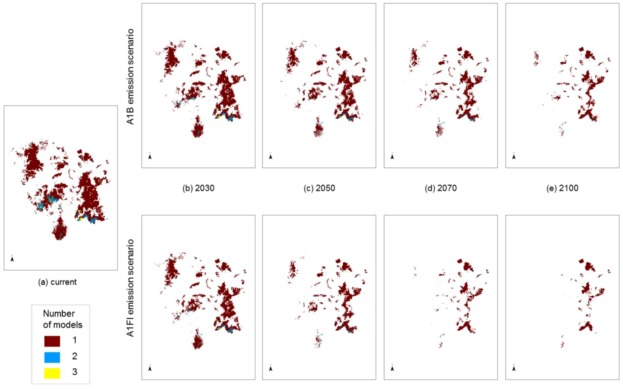
Modelled potential habitat for five threatened *Macrozamia* species in the Darling Downs region of Queensland, Australia under **(a)** current (historic) climate or with increasing annual mean temperatures under A1B and A1FI emission scenarios predicted in **(b)** 2030, **(c)** 2050, **(d)** 2070 and **(e)** 2100. Colours refer to the number of overlapping species models at a location.

The degree to which the areas of predicted future habitat for each species coincide was examined by stacking individual species models on top of each other (Figure 5). Some areas are highlighted as potential habitat suitable for two or three *Macrozamia* species, perhaps suggesting that greater exchange has occurred between these species in the past. The inferred phylogenetic hypothesis for this group of species is that they share a common ancestral lineage, and this is reflected in genetic similarities, though not uniformity ([14]; Ingham, unpublished data). This hypothesis encompasses the concept that the ancestral lineage (*viz.* a ‘superspecies’) fragmented into populations that became further separated into metapopulations with restriction to refugial areas or with some local migration. Past climate changes are likely to have been the primary drivers of fragmentation of the ‘superspecies’ yielding the present isolated distributions, although it is unlikely to have been a single event, rather a recurring pattern of expansion and contraction for this group. Figure 5 also suggests decreasing overlap in predicted ranges through time. This increasing isolation coupled with inbreeding and increasing genetic drift between members of the ‘superspecies’ has resulted in a group of similar species with relatively minor morphological and genetic differences. As such, they can be interpreted as an example of ‘non-radiation’, where there is a low lineage, diversification rate (*i.e.*, unsuccessful in terms of species production and ecological/morphological diversification) [46].

The future survival and role of specialized mutualist pollinators (*Tranes* beetles) [31,32,33,47,48,49] is a further compounding biological feature. The impacts of increasing annual mean temperature on these *Tranes* spp. beetles is largely unknown. Energetic rewards via thermogenic cycad cones may be reduced as ambient temperatures increase, particularly in the evenings, when pollination occurs. Temperature plays a primary role in controlling all stages of plant reproduction [50]; however, its role in controlling thermogenesis and its cues in cycad cones remains unclear. Roemer *et al.* [48,49] found that ambient temperatures are important in the initiation, magnitude and timing of cone temperature oscillations for several *Macrozamia* cycads (*M. macleayi*, *M. lucida*). Increasing ambient temperatures trigger thermogenic events in these species, so an increase in these temperatures may result in earlier coning cycles for these Macrozamias; whether the beetles can adapt to these changes remains to be seen. 

If the four critical questions by Schemske *et al.* [51] about species viability are addressed for these species, in conjunction with the conclusions reached for the biologically similar cycad *M. platyrhachis* [23], then the projected scenarios of temperature increases can be responded to thus:

**Question 1 - Biological status of the species**

Are individuals and populations of the species increasing, decreasing or stable? 

**Likely to decrease**. 

**Question 2 - Life history**

What stages are limiting population growth and species persistence? (a) Lack of seed dispersal, (b) poor recruitment as exhibited by extremely low current seedling survival and (c) adult plant dependence on a specific insect pollination mutualism for reproduction make these species highly sensitive to disturbances. 

**All three features are likely to be impacted negatively**.

**Question 3 - Biological causes of variation in life history**

What causes have the most demographic impact? (a) Lack of dispersal agents result in short (<100 m) or no seed dispersal. This limits range expansion and also results in inbreeding depression due to recruitment of siblings. (b) The long generation time (probably 20–25 years, 60–80 years for three generations) and the highly unpredictable nature of coning events likely contributes to variability in pollinator populations, fertilization and population recruitment. 

**Negative impact predicted.**

**Question 4 - Geographical and physical constraints**

Is there room to move? The species are restricted to areas of unique geology and topology in Queensland and are not likely to colonize outside of these areas. There are large areas that appear to be suitable habitat within the known area of occurrence, but lack of seed dispersal appears to limit expansion outside the population boundaries. 

**Unlikely to be able to move and in most instances, nowhere to move to; particularly in areas where clearing of vegetation for agriculture has occurred or is ongoing.**

## 4. Conclusions

An increase in ambient temperatures as predicted under certain climate models is likely to have an adverse effect on the five species of threatened *Macrozamia* cycads discussed. The models indicate that the area of suitable habitat will decrease and move upslope, thus further restricting species and populations into smaller and smaller refugial areas. The models also indicate that most of the species are already in refugial areas. Biological attributes of the cycads, such as limited dispersal that is controlling distribution, slow generational turnover and obligate pollination mutualisms (beetle pollination and thermogenic cones) are already ‘imprisoning’ the populations in refugia. In most instances, it is unlikely that the fragmented populations of either the individual species or the overall ‘superspecies’ will merge together under these climate prediction scenarios, nor will they be able to move at the rate necessary to keep up with change. 

Long term conservation of these ‘flagship’ species and the unique pollination mutualisms associated with them will involve careful monitoring of the health of individual populations, together with specialized management of the ‘core’ populations in refugial areas. Maintaining the overall assemblage of populations is likely to prove difficult, and comprehensive knowledge of individual population genetics is essential if informed decisions about species, population or individual rescue with subsequent ‘assistance’, such as augmentation, are to be successful. As individual adult plants are long-lived and resilient to the extremes of environmental variables, it is likely that they may persist in these areas for a long time, albeit as the ‘living dead’. 
